# Genome-Wide Identification and Analysis of the *TIFY* Gene Family in Grape

**DOI:** 10.1371/journal.pone.0044465

**Published:** 2012-09-11

**Authors:** Yucheng Zhang, Min Gao, Stacy D. Singer, Zhangjun Fei, Hua Wang, Xiping Wang

**Affiliations:** 1 College of Horticulture, Northwest A&F University, Key Laboratory of Horticultural Plant Biology and Germplasm Innovation in Northwest China, Ministry of Agriculture, Yangling, Shaanxi, P. R. China; 2 College of Enology, Northwest A&F University, Shaanxi Engineering Research Center for Viti-Viniculture, Yangling, Shaanxi, P. R. China; 3 State Key Laboratory of Crop Stress Biology in Arid Areas (Northwest A&F University), Yangling, Shaanxi, P. R. China; 4 Department of Agricultural, Food and Nutritional Science, University of Alberta, Edmonton, Alberta, Canada; 5 Boyce Thompson Institute for Plant Research, Cornell University, Ithaca, New York, United States of America; 6 USDA Robert W. Holley Center for Agriculture and Health, Ithaca, New York, United States of America; Purdue University, United States of America

## Abstract

**Background:**

The *TIFY* gene family constitutes a plant-specific group of genes with a broad range of functions. This family encodes four subfamilies of proteins, including ZML, TIFY, PPD and JASMONATE ZIM-Domain (JAZ) proteins. JAZ proteins are targets of the SCF^COI1^ complex, and function as negative regulators in the JA signaling pathway. Recently, it has been reported in both *Arabidopsis* and rice that *TIFY* genes, and especially *JAZ* genes, may be involved in plant defense against insect feeding, wounding, pathogens and abiotic stresses. Nonetheless, knowledge concerning the specific expression patterns and evolutionary history of plant *TIFY* family members is limited, especially in a woody species such as grape.

**Methodology/Principal Findings:**

A total of two *TIFY*, four *ZML*, two *PPD* and 11 *JAZ* genes were identified in the *Vitis vinifera* genome. Phylogenetic analysis of TIFY protein sequences from grape, *Arabidopsis* and rice indicated that the grape TIFY proteins are more closely related to those of *Arabidopsis* than those of rice. Both segmental and tandem duplication events have been major contributors to the expansion of the grape *TIFY* family. In addition, synteny analysis between grape and *Arabidopsis* demonstrated that homologues of several grape *TIFY* genes were found in the corresponding syntenic blocks of *Arabidopsis*, suggesting that these genes arose before the divergence of lineages that led to grape and *Arabidopsis*. Analyses of microarray and quantitative real-time RT-PCR expression data revealed that grape *TIFY* genes are not a major player in the defense against biotrophic pathogens or viruses. However, many of these genes were responsive to JA and ABA, but not SA or ET.

**Conclusion:**

The genome-wide identification, evolutionary and expression analyses of grape *TIFY* genes should facilitate further research of this gene family and provide new insights regarding their evolutionary history and regulatory control.

## Introduction

TIFY proteins comprise a plant-specific family of putative transcription factors that are increasingly believed to play an important role in stress response. This family owes their name to a conserved motif (TIF[F/Y]XG) located within an approximately 36 amino acid long TIFY domain and can be divided into four groups based on both phylogenetic and structural analyses [1], [2]. While all TIFY proteins bear a TIFY domain, those in the ZML subfamily, including ZIM (Zinc-finger expressed in Inflorescence Meristem) and ZIM-like (ZML) proteins, also contain both a C2C2-GATA zinc-finger DNA-binding domain and a CCT domain (CONSTANS, CO-like, TOC1). Conversely, proteins from both PEAPOD (PPD) and JAZ subfamilies lack GATA and CCT domains [3]. Interestingly, in addition to the TIFY domain, the JAZ subfamily also contain a conserved sequence of approximately 27 amino acids near their C-terminus, referred to as the Jas motif, which is similar in sequence to the N-terminal portion of the CCT domain [3] and bears the characteristic motif SLX_2_FX_2_KRX_2_RX_5_PY [4]. PPD proteins, on the other hand, bear a unique N-terminal PPD domain, as well as a divergent Jas motif that lacks the conserved PY at its C-terminus [3]. Finally, proteins from the TIFY subfamily contain only the TIFY domain [4].

While there is a general paucity of information concerning this gene family in the majority of plant species, information regarding the functions of several *TIFY* genes is beginning to accumulate in *Arabidopsis*. For example, *AtTIFY1* (*ZIM*) has been found to play a role in petiole and hypocotyl elongation [5], whereas *AtTIFY4a* (*PPD1*) and *AtTIFY4b* (*PPD2*) are involved in the coordination of leaf growth [6]. Perhaps the most well-characterized members of this family include the *JAZ* genes, which are gaining intense interest due to their apparent key role in the jasmonic acid pathway [7]–[9].

Plants are exposed to a range of both abiotic and biotic stress during their life-cycles. Small signaling molecules, such as jasmonic acid (JA), salicylic acid (SA), ethylene (ET) and abscisic acid (ABA), mediate plant responses to defend against stress and are thus essential for their survival in nature. Jasmonates, including JA and its bioactive derivatives, are key regulators of plant responses to both biotic stress, such as wounding, pathogen infection and insect attack, as well as abiotic stress, such as drought and ozone exposure [10]. Furthermore, in healthy, unwounded plant tissue, jasmonates also play a broad role in the control of various important developmental processes, including root growth, seed germination, tendril coiling, flower development and senescence [11].

As is the case for many other plant hormones, much of our knowledge concerning JA function has been derived from the characterization of *Arabidopsis* mutants that are deficient in JA synthesis or perception [3]. For example, the *Arabidopsis coi1* mutant, which was discovered in a forward genetic screen designed to identify mutations that confer resistance to coronatine-inhibited root elongation, is deficient in all jasmonate responses, indicating that *COI1* is a key regulator of JA signaling [12],[13]. Subsequent studies demonstrated that *COI1* encodes an F-box protein [13], which is a component of the E3-type ubiquitin ligase SCF (Skp/Cullin/F-box) complex. This discovery led to the suggestion that ubiquitination of specific target proteins by the SCF^COI1^ complex, along with their subsequent degradation, is likely pivotal for the activation of JA signaling and responses [14]. While COI1 target proteins remained elusive in extensive initial studies, in 2007 three independent research groups almost simultaneously identified JAZ proteins as fulfilling this role [7]–[9].

In cells containing low levels of bioactive jasmonates, JAZ proteins repress the activity of positive transcription factors (e.g. MYC2 and MYC3) involved in the expression of early response genes [2], [8], [9], [15]. Both developmental and environmental cues can induce plant cells to accumulate bioactive jasmonates, which causes the induction of SCF^COI1^-mediated degradation of JAZ proteins and the de-repression of transcription factors such as MYC2 [3]. Interestingly, JA treatment and/or environmental stress conditions also rapidly trigger the expression of *JAZ* genes, indicating that JA-induced *JAZ* expression may constitute a negative feedback loop that replenishes the JAZ protein pool and dampens the response to JA [7], [8], [16]. Although these findings uncovered the mechanism whereby plants sense and respond to jasmonates, it remains unclear how multiple JA-regulated cues are translated into specific responses. Differential regulation of *JAZ* gene expression is one possible mechanism for such fine-tuning of JA responses [17]. Indeed, transcriptional analysis of *JAZ* genes in response to JA treatment, herbivory, wounding, *Pseudomonas syringae* infection, and environmental stress such as drought, low temperature and salinity, has recently provided evidence of such differential induction of *JAZ* expression in response to these stimuli [17]–[19].

Grapevine (*Vitis vinifera*) is economically the most important perennial fruit crop worldwide. Both biotic and abiotic stress cause significant losses in grape yield and reduce berry quality. Since jasmonates play a critical role in modulating plant defenses [20], a better understanding of JA-mediated processes that contribute to grape stress tolerance would be of significant value. The release of the grape genome has allowed us to carry out a genome-wide identification and analysis of the *TIFY* gene family in this woody species. In this study, we identified two *TIFY*, four *ZML*, two *PPD* and 11 *JAZ* genes in the *V. vinifera* genome. In addition, phylogenetic and syntenic analyses revealed that both segmental and tandem duplication events have contributed to the evolution of the grape *TIFY* gene family. Since a systematic analysis of the differential regulation of *TIFY* gene expression under stress conditions that are relevant to grapes may provide insight into the mechanism behind stress defense in this genus, we further analyzed the expression profiles of a selection of grape *TIFY* genes under various abiotic and biotic stresses, as well as in response to different phytohormone treatments. This was carried out through the mining of publicly available microarray datasets, as well as quantitative real-time RT-PCR assays. The results obtained should provide a foundation for further evolutionary and functional characterization of *TIFY* genes in plants and yield another piece of vital information for the potential future improvement of plant stress tolerance, possibly through the manipulation of stress-related gene expression.

## Results

### Genome-wide Identification of *TIFY* Genes in Grape

A hidden Markov model (HMM) profile of the TIFY domain, as well as Jas and CCT motifs, were extracted from Pfam (accession numbers PF06200, PF09425 and PF06203, respectively). Based on this profile, an HMM algorithm (HMMER) was utilized to screen protein sequence data from the Grape Genome Database in an attempt to identify putative grape TIFY proteins. Nineteen grape proteins containing the TIFY domain, 13 proteins containing both a TIFY domain and a Jas motif, and 4 proteins containing both a TIFY domain and a CCT motif were detected using this method (Table 1). In order to confirm these results and further classify these proteins, the Pfam web server was used to examine their conserved domains. While all 19 proteins were found to contain a TIFY domain, the four proteins containing both a TIFY domain and a CCT motif were also found to bear a C2C2-GATA zinc-finger, and were thus predicted to belong to the ZML subfamily. Among the 13 grape TIFY proteins that contained a Jas motif, two lacked the conserved PY motif at their C-termini, which is characteristic of PPD proteins [3], and also included a PPD domain, which indicates that they are in fact PPD proteins. The remaining two proteins contained only a TIFY domain, and were therefore classified as members of the TIFY subfamily.

**Table 1:** Grape *TIFY* genes.

Gene ID	Accession No.	Chrom	Gene locus ID	CDS (bp)	ORF (aa)
VvJAZ1	XM_002284819	1	GSVIVG01011679001	1155	384
VvJAZ2	XM_002262714	1	GSVIVG01000967001	639	212
VvJAZ3	XM_003634778	4	GSVIVG01007188001	297	98
VvJAZ4	XM_002272327	9	GSVIVG01016721001	861	268
VvJAZ5	XM_002277733	10	GSVIVG01021514001	384	127
VvJAZ6	XM_002277769	10	GSVIVG01021516001	384	127
VvJAZ7	XM_002277916	10	GSVIVG01021518001	432	143
VvJAZ8	CBI30922	10	GSVIVG01021519001	393	130
VvJAZ9	XM_002277121	11	GSVIVG01015042001	807	268
VvJAZ10	XM_002263220	12	GSVIVG01023256001	702	233
VvJAZ11	XM_002282652	17	GSVIVG01008453001	1107	368
VvPPD1	XM_002279284	5	GSVIVG01018038001	978	325
VvPPD2	CBI25038	unknown	GSVIVG01003113001	1026	341
VvZML1	XM_002270325	3	GSVIVG01012518001	909	302
VvZML2	XM_002263671	9	GSVIVG01029593001	900	299
VvZML3	XM_002283717	18	GSVIVG01009197001	885	294
VvZML4	XM_002283702	18	GSVIVG01009198001	1107	368
VvTIFY1	XM_002268836	3	GSVIVG01012522001	534	177
VvTIFY2	XM_002282380	4	GSVIVG01035797001	1326	441

Taken together, we identified two *TIFY*, four *ZML*, two *PPD* and 11 *JAZ* genes. The grape *TIFY* genes were designated sequentially from *VvTIFY1* to *VvTIFY2*, *VvZML1* to *VvZML4*, *VvPPD1* to *VvPPD2*, and *VvJAZ1* to *VvJAZ11*, according to their genomic locations in the present study (Table 1). Among the 19 grape *TIFY* genes identified, nine were also supported by cDNA sequences that comprised full-length coding regions (corresponding GenBank accession numbers are *VvJAZ1*: FQ390867.1; *VvJAZ6*: FQ379446.1; *VvJAZ7*: FQ388100.1; *VvJAZ9*: FQ382107.1; *VvJAZ10*: FQ387210.1; *Vv JAZ11*: FQ382029; *VvZML1*: FQ379339.1and *VvZML2*: FQ393169.1; *VvTIFY2*:FQ382997.1) and 18 (including nine for which no cDNA evidence was available) were supported by at least one grape EST sequence. Therefore, only one grape *TIFY* gene (*VvPPD1*) lacked both EST and mRNA sequence support. Since the main focus of this study is to investigate TIFY members bearing either CCT or Jas motifs, the two *TIFY* subfamily genes (*VvTIFY1* and *VvTIFY2*) containing only a TIFY domain were not analyzed further.

### Phylogenetic analysis of *ZML*, *PPD* and *JAZ* genes from three plant species

**Figure 1:**
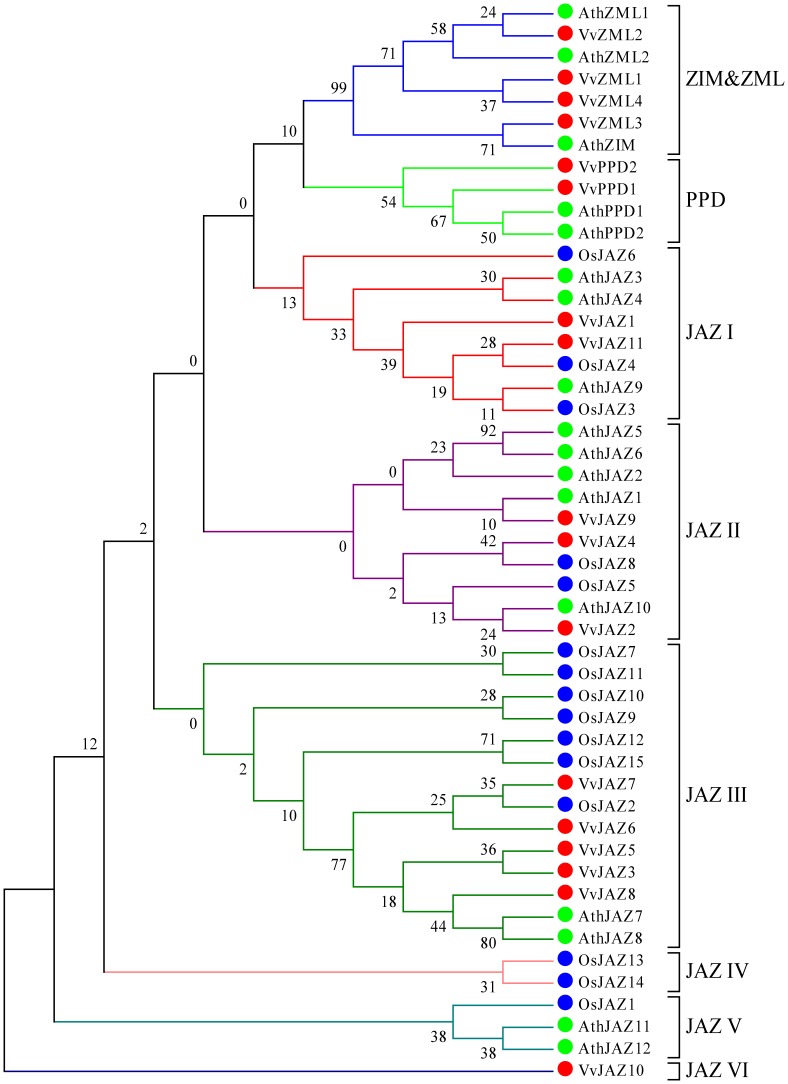
Phylogenetic analysis of grape, *Arabidopsis* and rice TIFY proteins. Phylogenetic tree was constructed with TIFY protein sequences from *V. vinifera* (Vv), *O. sativa* (Os) and *A. thaliana* (Ath).

Protein sequences derived from the *ZML*, *PPD* and *JAZ* nucleotide sequences identified in *V. vinifera*, along with TIFY protein sequences from *A. thaliana* [1] and *O. sativa* [18], were used to construct a phylogenetic tree (Fig. 1). Among the 17 analyzed grape *TIFY* genes, 11 (*VvZML2*, *VvZML1*, *VvZML4*, *VvZML3*, *VvPPD2*, *VvPPD1*, *VvJAZ9*, *VvJAZ2*, *VvJAZ3*, *VvJAZ5* and *VvJAZ8*) were grouped together with *Arabidopsis TIFY* genes rather than rice genes, indicating that the majority of *V. vinifera TIFY* genes were more closely related to those of *Arabidopsis* than those of rice, which is consistent with the fact that both grape and *Arabidopsis* are eudicots and diverged more recently from a common ancestor than from the lineage leading to monocots. The plant TIFY proteins analyzed here were classified into eight groups based on the phylogenetic tree. ZIM and ZML proteins were clustered into one group, PPD proteins comprised a second group, and JAZ proteins were divided into six clades (I to VI), indicating a broader phylogenetic relationship within this subset of genes.

Although evolutionary relationship could not be clarified for all proteins, some interesting observations were noted. VvJAZ10, for example, which alone constituted the JAZ VI clade, was phylogenetically the most divergent member of the JAZ proteins. Both the JAZ VI and JAZ IV clades only consisted of JAZ proteins from rice or grape, respectively, indicating that these genes may have undergone significant mutation/loss following the split between lineages leading to monocots and eudictos. Three of the JAZ clades (I, II and III) were composed of sequences from *Arabidopsis*, grape, and rice. The JAZ I clade contained similar numbers of genes from each species, suggesting that major expansion/contraction in this clade has not occurred since the divergence between eudicots (*Arabidopsis* and grape) and monocots (rice). Conversely, in the JAZ II and JAZ III clades, the number of genes from each of the three species differed widely, indicating that expansion/contraction occurred after the separation of each lineage.

### Sequence Comparison of Grape *ZML*, *PPD* and *JAZ* Genes

**Figure 2:**
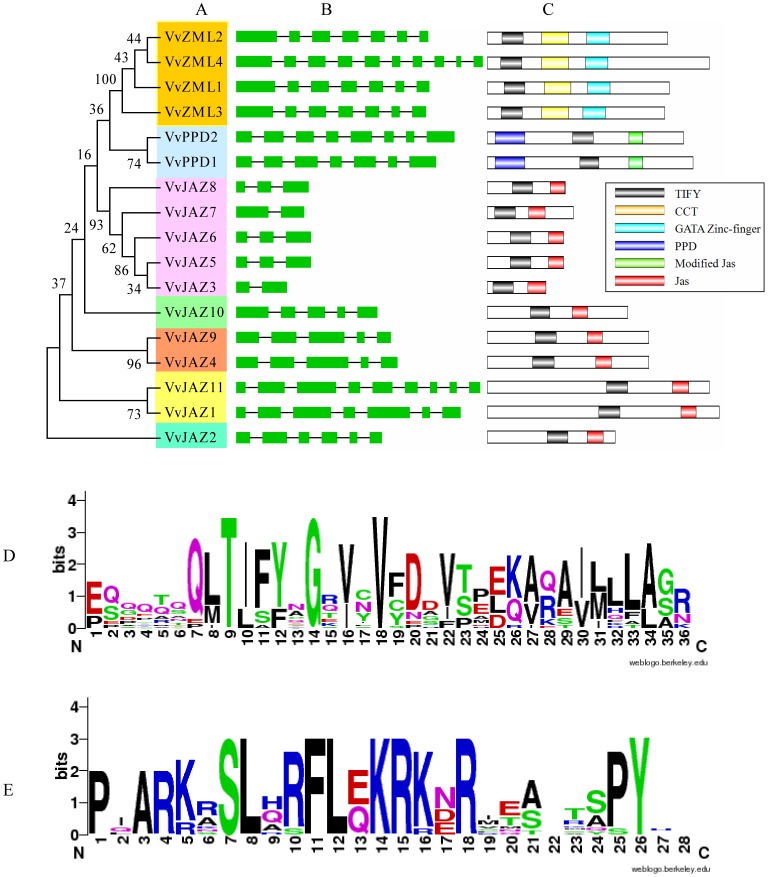
The ZML, PPD and JAZ protein subfamilies in grape. (A) Phylogenetic analysis of grape ZML, PPD and JAZ proteins. Numbers above or below branches of the tree indicate bootstrap values. (B) Exon/intron structures of grape *ZML, PPD* and *JAZ* genes. Only the exons, represented by green boxes, are drawn to scale. Black lines connecting two exons represent introns. (C) The distribution of conserved domains within grape ZML, PPD and JAZ proteins. The relative positions of each conserved domain within each protein are shown in color. (D) Sequence logo of the TIFY (D) and Jas (E) domains from grape TIFY proteins.

Phylogenetic analysis was also carried out using only the amino acid sequences of the 17 grape *TIFY* genes identified here (Fig. 2a). The topology was similar to that of the phylogenetic tree constructed using TIFY sequences from the three plant species (Fig. 1) and TIFY proteins from the same family tended to cluster together with JAZ proteins classified into five distinct groups (JAZ I, II, III, V and VI). One exception was the protein VvJAZ2, which had been grouped within the JAZ II clade in the multi-species analysis, but in the grape analysis was not included in any of the JAZ clades and was instead the most divergent member of the JAZ subfamily.

Exon/intron structure can also be used to provide additional evidence to support phylogenetic groupings [21] as this type of divergence often plays a key role in the evolution of gene families. Therefore, the exon/intron structures of the grape *JAZ*, *ZML* and *PPD* genes were examined (Fig. 2b) to gain further insight into their possible gene structural evolution. Our results indicated a strong correlation between their phylogeny and exon/intron structure, and genes that clustered together generally possessed a similar gene structure. Indeed, three sets of genes (*VvZML2*/*VvZML1*/*VvZM3*, *VvJAZ8*/*VvJAZ6*/*VvJAZ5* and *VvJAZ9*/*VvJAZ4*) comprised the exact same number of exons with nearly identical exon length, respectively (Fig. 2b), indicating that these *TIFY* genes may be the products of duplication events. Nonetheless, we did identify intron/exon loss/gain within several *TIFY* gene clades. For example, *VvZML4* was made up of 11 exons compared to the 7 contained by all other *VvZML* genes, indicating that it may have acquired four additional exons during evolution. Conversely, *VvJAZ7* appears to have lost its first intron in the course of its evolutionary history.

To provide further confirmation of the evolutionary relationships among the grape *TIFY* genes, we also visualized the distribution of their conserved domains (Fig. 2c). Although the number of amino acids of grape TIFY protein sequences varied from 98 to 441 (Table 1), proteins that clustered together tended to contain the same number of amino acids and a similar distribution of conserved domains. These results were consistent with the exon/intron structure analysis: members in different clades showed a great degree of sequence divergence, whereas members in the same clade bore a close relationship.

In order to investigate the level of conservation of the TIFY domain in all 17 grape proteins analyzed, as well as the Jas motif in JAZ proteins, sequence logos were constructed using the WebLogo program (http://weblogo.berkeley.edu). Results revealed that TIFY domains were not well conserved, except within the TIF [F/Y] XG region and several other amino acid sites (Fig. 2d), whereas the Jas motif was highly conserved with 100% identity of amino acids located at sites 1, 3, 4, 7, 8, 11, 12, 14, 15, 18, 26 (Fig. 2e).

### Expansion Patterns of *ZML*, *PPD* and *JAZ* Families in Grape

**Figure 3:**
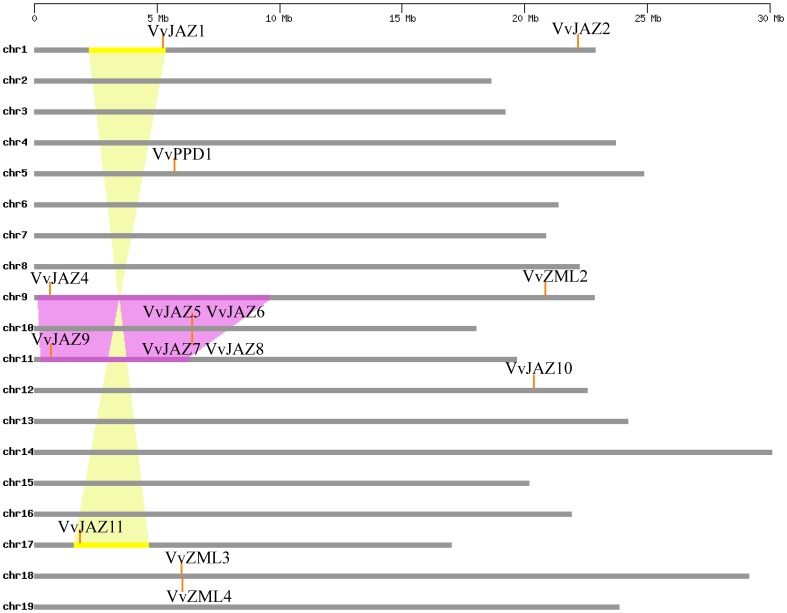
Distribution and synteny of *ZML*, *PPD* and *JAZ* **genes on grape chromosomes.** Chromosomes 1–19 (chr1–19) are depicted as horizontal gray bars. *ZML*, *PPD* and *JAZ* genes are indicated by vertical orange lines. Colored bars denote syntenic regions of the grape genome; a twisted colored bar indicates that syntenic regions are in opposite orientations.

Segmental and tandem duplications have been suggested to be two of the main causes of gene family expansion in plants [22]. To determine whether this has been the case for the grape *TIFY* gene family, we compared the chromosomal locations of fourteen grape *TIFY* genes (the chromosomal locations of *VvPPD2*, *VvZML1* and *VvJAZ3* are unknown and were therefore not included in this portion of the study). We identified one *JAZ* tandem duplication cluster (*VvJAZ5*/*VvJAZ6*/*VvJAZ7*/*VvJAZ8*), as well as one *ZML* tandem duplication cluster (*VvZML3*/*VvZML4*), on grape chromosomes 10 and 18, respectively (Fig. 3). We then examined duplicated blocks within the grape genome and found that four grape *JAZ* genes (*VvJAZ1*/*VvJAZ11* and *VvJAZ4*/*VvJAZ9*) were located in two pairs of duplicated genome regions (Fig. 3). In summary, ten of 14 grape *TIFY* genes were associated with either segmental or tandem duplication events, indicating that such duplications have likely played important roles in the expansion of this gene family in grape.

### Evolutionary Relationship of Grape and *Arabidopsis ZML*, *PPD* and *JAZ* Genes

**Figure 4:**
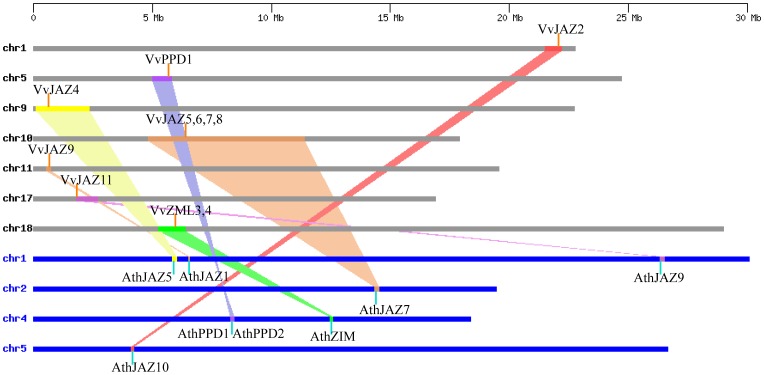
Synteny analysis of *ZML*, *PPD* and *JAZ* genes between grape and *Arabidopsis*. Grape and *Arabidopsis* chromosomes are depicted as horizontal gray and blue bars, respectively. Grape and *Arabidopsis TIFY* genes are indicated by vertical orange and blue lines, respectively. Colored bars denote syntenic regions between grape and *Arabidopsis* chromosomes; a twisted colored bar indicates that the syntenic regions are in opposite orientations.

The comparison of gene sequences among various plant genomes, as well as within each genome, provides the information necessary to reconstruct the evolutionary history of each gene [23]. Furthermore, genomic comparison is a relatively rapid way to transfer genomic knowledge acquired in one taxon to a less-studied taxon [24]. Therefore, to further explore the origin and evolutionary history of the grape *ZML*, *PPD* and *JAZ* genes, we generated a comparative syntenic map between grape and *Arabidopsis* genomes (Fig. 4). Since *Arabidopsis* is the most important model plant species and the functions of some *Arabidopsis TIFY* genes have been well-characterized, we were able to infer the functions of several grape *TIFYs* based on their *Arabidopsis* homologues through comparative genomic analyses.

Large-scale syntenies containing *TIFY* gene orthologues from both grape and *Arabidopsis* were identified that included two grape *ZML* genes (*VvZML3* and *VvZML4*), one grape *PPD* gene (*VvPPD1*), and eight grape *JAZ* genes (*VvJAZ2*, *VvJAZ4, VvJAZ5, VvJAZ6, VvJAZ7, VvJAZ8, VvJAZ9 and VvJAZ11)* (Fig. 4), indicating the majority of grape *TIFY* genes appeared to share a common ancestor with their *Arabidopsis TIFY* counterparts. With regard to single grape-to-*Arabidopsis TIFY* gene correspondences, the syntenies were unambiguous and included two orthologue pairs: *VvJAZ2*-*AthJAZ10* and *VvJAZ11*-*AthJAZ9.* A more challenging aspect of the syntenic interpretation included cases where single grape genes corresponded to *Arabidopsis* tandem duplications or grape tandem duplications corresponded to single *Arabidopsis* genes. These included *VvJAZ5*/*VvJAZ6*/*VvJAZ7*/*VvJAZ8*-*AthJAZ7*, *VvPPD1*-*AthPPD1*/*AthPPD2* and *VvZML3*/*VvZML4*-*AthZIM*. Finally, one case was identified where two duplicated grape genes corresponded to two *Arabidopsis* genes (*VvJAZ4*/*VvJAZ9*-*AthJAZ1*/*AthJAZ5*). In such an instance, it is not possible to elucidate whether the segmental duplications occurred prior to or after divergence from a common ancestor.

### Expression Profiles of a Selection of Grape *TIFY* Genes

**Figure 5:**
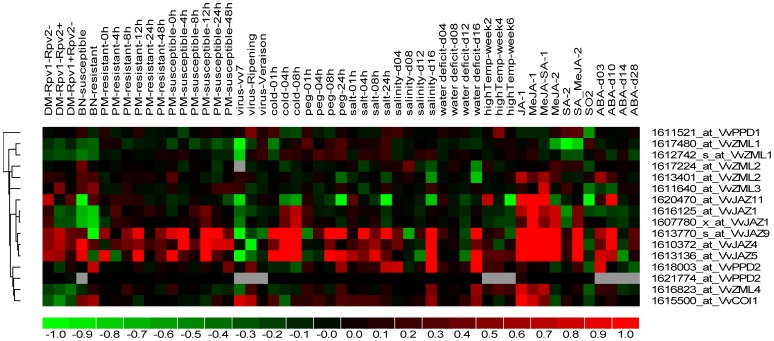
Hierarchical clustering of a selection of *TIFY* genes. Details of the experimental conditions are provided in Table S1. Log2 based fold-changes were used to create the heatmap. Differences in gene expression changes are shown in color as per the lower scale.

In the present study, we investigated the response of grape *ZML*, *PPD* and *JAZ* genes to various abiotic and biotic stress conditions, as well as hormone treatments, by mining publicly available grape microarray datasets. A total of 12 experiments containing 257 hybridizations from grape genome arrays were obtained and subjected to manual curation, and 53 comparisons between various different experimental conditions were constructed. From these results, we identified 11 grape *TIFY* transcripts corresponding to 15 probe sets, including *VvZML1*, *VvZML2*, *VvZML3*, *VvZML4*, *VvPPD1*, *VvPPD2*, *VvJAZ1*, *VvJAZ4*, *VvJAZ5*, *VvJAZ9* and *VvJAZ11*. As JAZ proteins are known targets of the SCF^COI1^ complex, we also investigated the correlation between *JAZ* and *COI1* gene expression. Therefore, we also identified a probe set for the grape *COI1* gene. Detailed expression profiles of the grape *TIFY* and *COI1* genes are provided in Table S1. In addition, a heat map representation of the expression profiles of these genes is shown in Fig. 5, revealing that several grape *TIFY* genes (mainly *JAZ* genes) were highly responsive to certain types of abiotic stress and hormone treatments.

#### Abiotic stress

Abiotic stress such as drought, extreme temperatures, and salinity adversely affects plant growth and crop productivity. The microarray data analyzed here included hybridizations generated from plants exposed to polyethylene glycol (PEG), which induces drought and has been used widely to stimulate a drought response [25], [26], as well as cold, high temperatures, high salinity and water-deficit stress [27], [28]. Our analysis of publicly available microarray datasets indicated that the expression of the majority of the 11 grape *TIFY* genes analyzed, as well as the *COI1* gene, were differentially expressed in at least one of the four osmotic treatments, including short-term PEG (1–24 h), short-term salinity (1–24 h), long-term salinity (4–16 days) and long-term water-deficit (4–16 days) (Table S1). Among the genes affected, eight (*VvPPD2*, *VvJAZ4*, *VvJAZ5*, *VvJAZ9*, *VvZML1*, *VvZML3*, *VvZML4* and *VvCOI1*) exhibited enhanced expression, whereas two (*VvJAZ11* and *VvZML2*) were down-regulated. Under cold conditions (5°C), four genes (*VvJAZ1*, *VvJAZ4*, *VvJAZ5* and *VvJAZ9*) demonstrated increased expression, while three (*VvJAZ11*, *VvZML1* and *VvACOI1*) exhibited decreased expression (Table S1). Interestingly, when subjected to heat stress, only *VvJAZ11* displayed the change in expression, with down-regulation (Table S1).

#### Biotic stress

*Plasmopara viticola* is the causal agent of downy mildew, one of the world’s most catastrophic and baffling diseases of grapevine [29]. Two QTLs, Rpv1 and Rpv2, located in chromosomes 12 and 18, respectively, were found to be responsible for the resistance to *P. viticola* in grape [30]. Our microarray data analysis revealed that in a grape line that is highly resistant to *P. viticola* (Rpv1−/Rpv2+), three of the 11 *TIFY* genes analyzed were differentially expressed upon inoculation with *P. viticola*, with two up-regulated (*VvZML3* and *VvJAZ9*) and one down-regulated (*VvZML4*). Conversely, in both partially resistant (Rpv1+/Rpv2−) and susceptible (Rpv1−/Rpv2−) lines, only *VvZML4* exhibited differential expression upon *P. viticola* infection in the form of down-regulation. In the case of *VvCOI1*, it only exhibited reduced expression in the highly resistant line (Rpv1−/Rpv2+) upon *P. viticola* infection.

Powdery mildew, caused by the obligate biotrophic fungus, *Uncinula necator* [Schw.] Burr., adversely affects vine growth, berry quality and grape production [31]. Microarray experiments were conducted previously to investigate any *U. necator*-induced changes in the transcriptome of *V. vinifera* ‘Cabernet sauvignon’ and the powdery mildew resistant *V. aestivalis* ‘Norton’ [31]. Array data indicated that the expression levels of all 11 *TIFY* genes analyzed, as well as *VvCOI1*, were not significantly altered upon infection with the fungus in either the disease-resistant *V. aestivalis* ‘Norton’ or the disease-susceptible *V. vinifera* ‘Cabernet sauvignon’ (Table S1).

In several grapevine growing countries such as France and Spain, *V. vinifera* is severely affected by Bois Noir, an emerging grapevine yellows disease caused by phytoplasmas, which are microscopic plant pathogens that are similar to bacteria, but much smaller and lacking cell walls [32]. Transcriptional changes in *V. vinifera* cultivars ‘Chardonnay’ (susceptible) and ‘Manzoni Bianco’ (moderately resistant) naturally infected with Bois Noir phytoplasma were analyzed previously [33]. In both grape cultivars, only *VvJAZ1* was down-regulated after infection (Table S1).

Among the more than 40 different viruses known to infect grapevines, leaf roll-associated closeterovirus-3 (GLRaV-3) is one of the most widespread [34]. *V. vinifera* cv. ‘Cabernet Sauvignon’ berry transcriptomes at two stages of development (veraison and ripening) infected with GLRaV-3 were analyzed previously [35] and our subsequent study indicated that expression of *VvPPD2* and *VvJAZ4* were up-regulated and down-regulated in ripening berries when infected with GLRaV-3, respectively. However, none of the *TIFY* genes showed significant changes in expression at veraison (Table S1). *VvCOI1* also exhibited enhanced expression upon GLRaV-3 infection in ripening berries, but not at veraison. Furthermore, data from a separate array experiment [36], indicated that none of the genes analyzed here exhibited significant alterations in expression upon GLRaV-3 infection in the leaves of two *V. vinifera* red wine cultivars (‘Carménère’ and ‘Cabernet Sauvignon’).

#### Hormone treatment

Analysis of expression data from red-skinned ‘Crimson Seedless’ grape (*V. vinifera* L. ) cell-suspension cultures exposed to JA, MeJA, or a combination of SA and MeJA [37], which are all crucial for biotic stress responses in plants [38], indicated that almost all *TIFY* genes analyzed here, as well as *VvCOI1*, were differentially expressed upon both JA and MeJA treatment, with the exception of *VvPPD2*, *VvZML3*, and *VvZML1*. Among the JA-responsive genes, all but *VvPPD1* were up-regulated. While *VvPPD2* was slightly up-regulated upon treatment with JA, its expression was not significantly altered upon MeJA treatment. However, in a separate study in which harvested grape berries were treated with SA, MeJA, SO_2_ or a combination of SA and MeJA [39], only three of the grape genes analyzed (*VvJAZ4*, *VvJAZ5* and *VvJAZ9*) were significantly up-regulated upon MeJA treatment, while *VvPPD1* was down-regulated upon SO_2_ treatment.

In the skin of grape berries treated with exogenous ABA [40], which is known to play a central role in the response of plants to various types of abiotic stress, six of the 11 *TIFY* genes analyzed exhibited altered levels of expression. Among them, five (*VvJAZ4*, *VvJAZ9*, *VvJAZ11*, *VvZML2* and *VvZML4*) were up-regulated, while *VvJAZ1* was down-regulated. Conversely, the expression of *VvCOI1* was not significantly altered upon ABA treatment.

**Figure 6:**
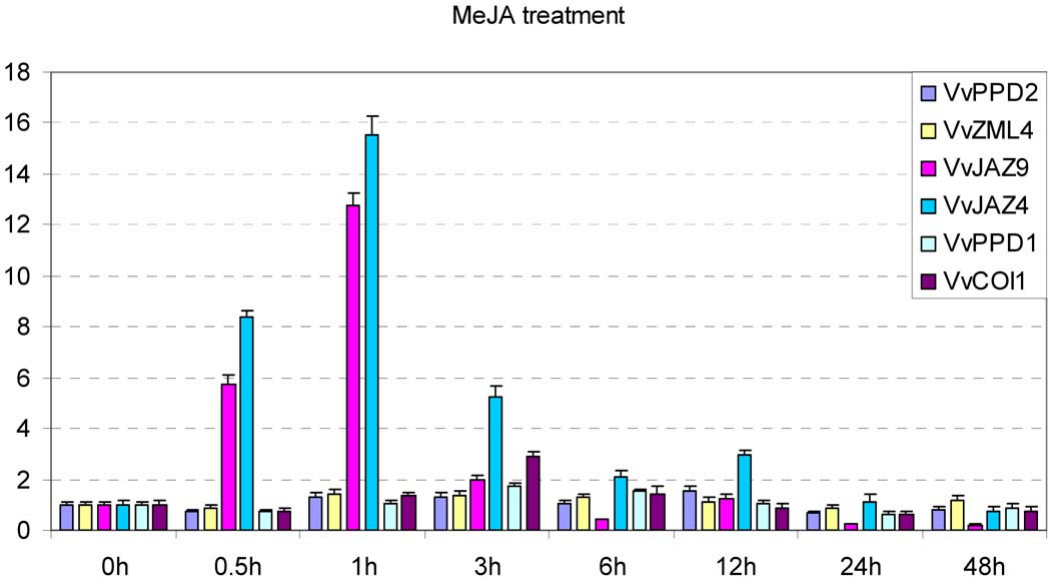
Expression levels of *TIFY* genes and *VvCOI1* following MeJA treatment in the leaves of Chinese wild *Vitis pseudoreticulata* ‘Hunan-1’. Grape *Actin1* was used as internal control for qRT-PCR and fold changes indicate expression level in treated leaves compared with negative control, which was set to 1. Mean values and SDs were obtained from three technical and three biological replicates.

**Figure 7:**
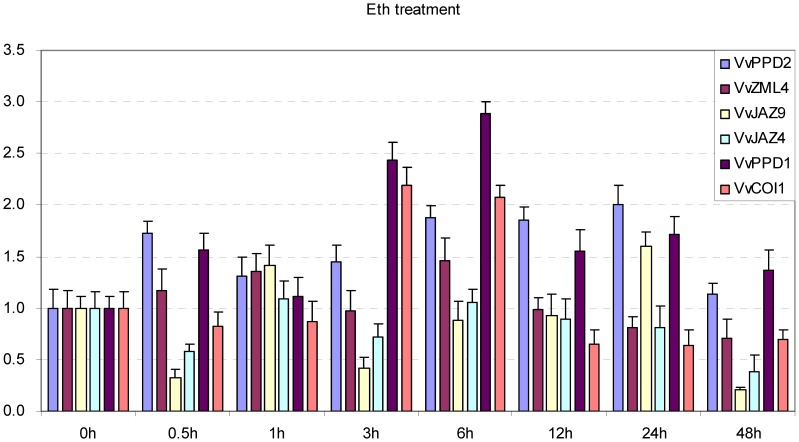
Expression levels of *TIFY* genes and *VvCOI1* following Eth treatment in the leaves of Chinese wild *Vitis pseudoreticulata* ‘Hunan-1’. Grape *Actin1* was used as internal control for qRT-PCR and fold changes indicate expression level in treated leaves compared with negative control, which was set to 1. Mean values and SDs were obtained from three technical and three biological replicates.

To confirm the results of these array analyses, we selected two genes (*VvJAZ4* and *VvJAZ9*) that were significantly up-regulated by JA, two genes (*VvZML4* and *VvPPD2*) that were slightly up-regulated by JA, and one gene (*VvPPD1*) that was slightly down-regulated by JA, as well as *VvCOI1*, and carried out quantitative real-time RT-PCR (qRT-PCR) assays to test expression in the leaves of Chinese wild *Vitis pseudoreticulata* ‘Hunan-1’ upon MeJA treatment (Fig. 6). Since the microarray data lacked ethylene (ET) treatment, we also investigated the expression of the six grape genes following treatment with this signaling molecule (Fig. 7). The results obtained were consistent with the array results in that both *VvJAZ4* and *VvJAZ9* were significantly induced by MeJA, and *VvCOI1* was also moderately up-regulated by this same hormone. In contrast, the expression of the remaining three genes (*VvZML4*, *VvPPD1* and *VvPPD2*) did not appear to be obviously altered by MeJA. Following ethylene treatment, we found that *VvPPD1*, *VvPPD2* and *VvCOI1* were moderately up-regulated, whereas no obvious changes were noted in the expression of the other three genes analyzed (*VvZML4*, *VvJAZ4* and *VvJAZ9*).

## Discussion

Members of the plant-specific TIFY family of putative transcription factors have recently been implicated in various stress responses within plants [17], [18], with the JAZ subfamily probably being the most well-characterized to date. However, virtually nothing is known about this family in woody species, such as grape. Since grapevine is one of the most important crops worldwide, and various forms of both biotic and abiotic stress can have an enormous impact on its production, further insight into stress-related responses in this genus could prove to be a significant asset. Therefore, we have sought to undertake the genome-wide identification of *TIFY* genes in grape, and provide clues regarding both their evolutionary history and expression diversity under various stress-related conditions.

### Grape *TIFY* Genes Diverged Early

*TIFY* homologues are found only in land plants and not in green algae or non-photosynthetic eukaryotes [1], indicating that this family originated after the transition of aquatic plants to land. The TIFY family has been found previously to comprise four major groups, including the ZML, TIFY, PPD and JAZ subfamilies [4]. In this study, we identified 19 *TIFY* genes within the grape genome (Table 1), among which two belong to the TIFY subfamily, four to the ZML subfamily (VvZML1–4), eleven to the JAZ subfamily (VvJAZ1–11) and two to the PPD subfamily (VvPPD1–2). It is worth noting that although VvJAZ11 lacked the PY signature, it was found to be a syntenic homolog of both VvJAZ1 and *Arabidopsis* AthJAZ9 (Fig. 3 and 4) and was therefore grouped into the JAZ family.

According to a previous study, seven *JAZ* genes were identified in the *V. vinifera* genome [2], which differs from the eleven *JAZ* genes identified in this study. This divergence is likely the result of the fact that the present study analyzed the 12X grape genome, while the previous study searched for *TIFY* genes in the 8X grape genome [2]. While a selection of grape *TIFY* genes were named based on their genome location previously [2], we have identified new *TIFY* members, and thus their annotation has been updated presently (Table 1).

All 11 *JAZ* genes that were identified in the grape genome shared only a relatively low level of nucleotide sequence identity and varied in length in terms of their corresponding proteins from 98 to 384 amino acids (Table 1). This high protein sequence variability among members of the JAZ subfamily has also been reported in *Arabidopsis* [1] and rice [18], indicating that these genes likely diverged early on during land plant evolution and have undergone significant mutations, and possibly functional divergence, since then. This subfamily of grape proteins was grouped into five separate clades (Fig. 2a), which correlates well with the findings of a previous study in which JAZ proteins from a variety of angiosperm species, including *V. vinifera*, were also found to fall within five clades [2].

In both the multi-species and single species phylogenetic trees generated here (Fig. 1 and 2a), it was clear that the two grape PPD proteins, were phylogenetically quite distant from JAZ proteins. Recently, genes belonging to the *ZML*, *JAZ* and *TIFY* subfamilies have been identified in the genome of the moss, *Physcomitrella patens*, which suggests that they diverged from one another very early on during the course of land plant evolution. In contrast, there is a distinct lack of *PPD* genes in *P. patens*, as well as in monocot species [2]. Since PPD genes are present in the non-seed vascular plant, *Selaginella moellendorffii* [2], it seems that they may have arisen more recently, following the divergence of vascular plants, and subsequently been lost in the monocot lineage.

### Tandem and Segmental Duplications Contributed to the Expansion of the Grape *TIFY* Gene Family

Gene duplication, including tandem, segmental, and whole genome duplication, has played an important role in the evolution of various organisms [41]. Since the grapevine genome has not undergone any recent whole genome duplication events [42], segmental and tandem duplications would be the two main causes of gene family expansions in grape. In this study, ten of 14 grape *TIFY* genes for which chromosomal locations were known were associated with either segmental or tandem duplication events, which is consistent with findings in rice that 16 of 20 *TIFY* genes were found to be located in either tandemly or segmentally duplicated regions [15]. Taken together, this implies that tandem and segmental duplication events likely played a central role in the expansion of the *TIFY* family in plants. Although the duplicated grape *TIFY* genes identified in this study have a common ancestor, we could not conclude from the work conducted here that they would have the same functions and expression patterns since duplicated genes, if they survive, tend to diverge in both their regulatory and coding regions during evolution, which often leads to paralogues that are functionally distinct [41].

### The Majority of Grape and *Arabidopsis TIFY* Genes are Syntenic Orthlogues

Comparative genomic analyses across different taxa allows the transfer of functional information from a taxon for which there is a better understanding of genome structure, function and/or evolution to another less-studied taxon [43]. Thus, the richness of gene functional information known for model plants such as *Arabidopsis* enables the inference of probable functions of their orthologous genes in diverse other plant taxa. Since the majority of grape and *Arabidopsis TIFY* genes are located in syntenic regions, and knowledge concerning grape *TIFY* genes is limited, we sought to infer functions of the grape *TIFY* genes based on their *Arabidopsis* counterparts. Previous studies have demonstrated that eight *Arabidopsis JAZ* genes (*JAZ1*, *JAZ2*, *JAZ5*, *JAZ6*, *JAZ7*, *JAZ8*, *JAZ9* and *JAZ10*) were responsive to JA [3], [8], among which, five (*JAZ1*, *JAZ5*, *JAZ7*, *JAZ9* and *JAZ10*) have grape syntenic orthlogues (Fig. 4) that were also responsive to JA in the present study (Table S1). In addition, other aspects of *Arabidopsis TIFY* gene function, including alternative splicing [3] and interaction with MYC2 [7], have been analyzed systematically; which allows the prediction of such aspects of the grape *TIFY* genes based on their *Arabidopsis* syntenic orthlogues.

Although three grape *TIFY* genes (*VvJAZ1*, *VvJAZ10* and *VvZML2*) could not be mapped into any syntenic blocks, this does not necessarily mean that these genes do not have orthologues in *Arabidopsis*. Instead, this could be explained by the fact that after the divergence of lineages that led to grape and *Arabidopsis*, their genomes have undergone multiple rounds of significant chromosomal rearrangement and fusions, followed by selective gene loss, which can severely obscure the identification of chromosomal syntenies [43].

### Divergence of Grape *TIFY* Gene Structure

Although several models for the evolution of genomes have been proposed from comparative genome analyses of model organisms [44]–[46], little attention has been paid to the gene structural evolution of duplicate gene families [47]. In fact, exon/intron diversification of gene family members has played an important role in the evolution of multiple gene families through three main types of mechanism: exon/intron gain/loss, exonization/pseudoexonization, and insertion/deletion [41]. In this study, it is clear from our analyses that grape *TIFY* genes within the same phylogenetic clade (Fig. 2a) that bear highly similar exon/intron structures (Fig. 2b) are the products of either segmental or tandem duplications (Fig. 3), which is consistent with findings in *Arabidopsis* [2] and rice [18]. Nevertheless, exon/intron gain/loss and divergence in exon/intron length were observed within the coding sequences of several of the grape *TIFY* genes, which could potentially lead to the generation of functionally distinct paralogues [41]. Interestingly, it has been reported that duplicated genes rarely diverge with respect to their biochemical function, but instead are limited to alterations in regulatory control [48]. However, further research is required to elucidate the specifics of any functional divergence between grape *TIFY* genes.

### The Majority of Grape *TIFY* Genes are not Responsive to Biotrophic Pathogens or Virus Infection

Plant response to biotic stresses, such as insect herbivory and pathogen infection, can be mediated by a variety of signaling molecules including JA, SA and ET [38], [49]. The *JAZ* subfamily of *TIFY* genes in particular have been implicated in JA responses, whereby jasmonates induce the SCF^COI1^-mediated degradation of JAZ proteins, resulting in the de-repression of transcription factors such as MYC2 and MYC3 [2], [8], [9], [15], while at the same time up-regulation of certain *JAZ* genes has been suggested to be a method of fine-tuning JA responses [3]. Interestingly, it has been shown that the majority of *Arabidopsis JAZ* genes are induced by *Pseudomonas syringae* infection [17], providing evidence to support the hypothesis that this subfamily of TIFY proteins, at least, are involved in plant pathogen resistance. However, in our study, only a minority of grape *TIFY* genes were slightly up-regulated following infection with *P. viticola* or the GLRaV-3 virus (TableS1), while infection with powdery mildew or Bois Noir phytoplasma resulted in no induction in any grape *TIFY* genes analyzed here.

This phenomenon may be explained by the fact that jasmonates mainly control plant resistance to necrotrophic pathogens, such as *Alternaria brassicicol*a and *Botrytis cinerea* [50]. It has been suggested that effective defense against biotrophic pathogens is largely due to programmed cell death in the host, and the associated activation of defense responses regulated by the SA–dependent pathway. In contrast, necrotrophic pathogens benefit from host cell death, so they are not limited by this or SA–dependent defenses, but rather by a different set of defense responses activated by JA and ET signaling [50]. None of the grape pathogens analyzed in our study was necrotrophic in nature, so they would not be likely to trigger activation of JA-dependent defense and *TIFY* gene induction. Though *P. syringae* is also a biotrophic pathogen, its virulence factor coronatine is an inducer of JA/ET signaling [50], which could explain why *Arabidopsis JAZ* genes were up-regulated upon infection with this organism [17].

### Grape *TIFY* Genes are Responsive to Several Forms of Abiotic Stress

Signaling molecules such as JA are not only involved in biotic stress responses, but also play important roles in the defense of plants against abiotic stresses, such as drought and salinity [20]. Recently, there has been evidence that *JAZ* genes from rice could be induced by various types of abiotic stresses, such as drought, salinity and low temperature [18]. In addition, over-expression of the *JAZ* gene *OsTIFY11a* in rice was found to improve stress tolerance [18]. In our study, we found that with the exception of *VvPPD1*, all of the grape *TIFY* genes analyzed were regulated by at least one type of abiotic stress (Table S1). The majority of the 11 grape *TIFY* genes analyzed were responsive to osmotic- and cold-stress, and all but one of the genes exhibiting responsiveness to cold was also regulated by drought or salinity, which implies that there may be crosstalk between these two types of stress pathways in plants. In contrast, only a single grape *TIFY* gene (*VvJAZ11*) exhibited a response to heat stress, which suggests that these genes may play a larger role in the former two types of abiotic stress than the latter.

### Grape *TIFY* Genes are Regulated by JA and ABA, but not SA or ET

There is abundant evidence supporting that JA treatment and environmental stresses rapidly trigger the expression of *JAZ* genes, which may moderate the response to JA [7], [8], [16]. In this study, we found that several grape *JAZ* genes were significantly up-regulated by both JA and MeJA treatments (Fig. 6, Table S1). In contrast, the expression of *PPD* and *ZML* genes was only slightly induced or not altered at all by the same treatments. Therefore, although *JAZ*, *ZML* and *PPD* genes all belong to *TIFY* family, their regulatory control appears to differ widely. Furthermore, our observation that grape *TIFY* genes were not responsive to SA or ET (Fig. 7, Table S1) further supports that grape defense against biotrophic pathogens, which are SA-dependent, were TIFY-independent.

ABA plays a key role in the ability of a plant to adapt to adverse environmental conditions, such as water-deficit, cold and salinity [51]. For example, in rice, four of 20 *TIFY* genes were found to be induced by ABA [18]. In the present study, six of the 11 grape abiotic stress-responsive *TIFY* genes analyzed were also found to be regulated by ABA. Since five of these genes were apparently not regulated by ABA (Table S1), it seems that both ABA-dependent and ABA-independent signaling pathways may regulate the expression of *TIFY* genes in grape.

### Grape *TIFY* Genes have Diverse Gene Expression Patterns

Based on our expression analyses of grape *TIFY* genes, it was apparent that although *JAZ*, *PPD*, and *ZML* genes all contained the TIFY domain and belonged to the TIFY protein family, their expression patterns differed widely, insinuating that these three groups of protein likely play distinct roles during plant development. Furthermore, the expression patterns of particular *JAZ* members often exhibited distinct differences. For example, while *VvJAZ11* was down-regulated by low temperature, PEG treatment and salinity, all remaining *JAZ* genes were up-regulated by at least one type of abiotic stress (Table S1). A similar phenomenon has also been observed in rice, where *JAZ* genes exhibited distinct expression patterns under abiotic stress and even JA treatment [18]. Since plant genomes generally contain a relatively large number of *TIFY* genes, this differential regulation of *TIFY* gene expression may be a mechanism by which stress responses are fine-tuned, although additional work is needed to confirm this hypothesis.

### The Correlation between Grape *JAZ* and *COI1* Genes

Although JAZ proteins are the targets of the SCF^COI1^ complex, our study indicated that the expression patterns of grape *JAZ* and *COI1* genes were not completely correlated. Under JA treatment and osmotic stress conditions (PEG, salinity and drought), both *VvCOI1* and the majority of *JAZ* genes analyzed here were up-regulated. However, while low temperatures triggered the expression of the majority of *JAZ* genes, *VvCOI1* was down-regulated. In addition, nearly all *JAZ* genes tested here were ABA-responsive, while ABA treatment did not significantly alter the expression of *VvCOI1*. These observations may be explained by the fact that although COI1 is required for JA responses, it also participates in other JA-independent pathways. For example, a recent study indicated that COI1 was involved in ET-induced inhibition of *Arabidopsis* root growth. Thus, when taken together, the present and previous studies suggest that since *COI1* is involved in other signaling pathways besides the JA-dependent, *JAZ* and *COI1* genes are not always concurrently expressed.

### Conclusion

The plant-specific *TIFY* gene family comprises four subfamilies, *ZML*, *TIFY*, *PPD* and *JAZ*. As the target proteins of the SCF^COI1^ complex and functioning as key components of the JA signaling pathway, JAZ proteins have gained widespread attention. Recently, significant progress has been made toward the identification and characterization of *TIFY* genes in model plants; however, little is known concerning this gene family in other plant species. In the present study we identified two *TIFY*, four *ZML*, two *PPD*, and 11 *JAZ* genes in the *V. vinifera* genome. The separation of the grape *JAZ* genes into five groups was mutually supported by their exon/intron structure, phylogeny, and the distribution of conserved domains. We further demonstrated that segmental and tandem duplications have contributed substantially to the expansion of grape *TIFY* gene family. Comparative synteny analysis between the *V. vinifera* and *Arabidopsis* genomes indicated that the majority of grape and *Arabidopsi*s *TIFY* genes were located in syntenic regions, which implies that these genes had common ancestors. Finally, we analyzed the expression profiles of 11 grape *TIFY* genes in response to various abiotic and biotic stress conditions, as well as hormone treatments. We found that the grape *TIFY* genes did not appear to play a major role in defense against biotrophic pathogens or viruses; however, a number of *TIFY* genes were responsive to JA and/or ABA, but not SA or ET. In addition, we also identified several grape *TIFY* genes that may potentially be involved in tolerance to environmental stresses. This information furthers our understanding of this group of genes in plants and provides a framework for future functional studies of the *TIFY* family in grape.

## Materials and Methods

### Identification and Annotation of Grape *TIFY* Genes

To identify members of the *TIFY* gene family in grape, previously identified *Arabidopsis* TIFY sequences were first submitted to the Pfam database (http://pfam.sanger.ac.uk) [52] to obtain the domain architecture of this family. The TIFY domain, Jas and CCT motifs were found to be represented by Pfam accession numbers PF06200, PF09425 and PF06203, respectively. Searches for each domain within the Grape Genome Database (12X; http://www.genoscope.cns.fr) were performed using HMMER [53] with an E-value <1e^-6^. To confirm results obtained using the HMMER algorithm, protein motifs were also queried against the Pfam database.

### Sequence Alignments and Phylogenetic Analyses

Multiple alignments of ZML, PPD, and JAZ protein sequences from *Vitis vinifera*, *Arabidopsis thaliana* [1] and *Oryza sativa* [18] were performed using the ClustalW program [54]. Phylogenetic trees were constructed with the MEGA 4.0 software using the maximum parsimony (MP) method and a bootstrap test that was replicated 1000 times [55].

### Exon/intron Structure Analysis of Grape *ZML*, *PPD* and *JAZ* Genes

The exon/intron structures of the grape *ZML*, *PPD* and *JAZ* genes were determined from alignments of their coding sequences with corresponding genomic sequences using the est2genome program, which aligns spliced mRNA sequences to the genome to obtain the exon/intron structure of genes [56]. A diagram of exon/intron structures was obtained using the online Gene Structure Display Server (GSDS: http://gsds.cbi.pku.edu.cn), which exhibited both exon position and gene length. Since the introns of several of the genes analyzed were relatively long, only the coding exons were drawn to scale.

### Tandem Duplication and Synteny Analysis

Tandem duplications of *ZML*, *PPD* and *JAZ* genes in the grape genome were predicted by determining their physical locations on individual chromosomes. Tandemly duplicated genes were defined as adjacent homologous genes on a single chromosome, with no more than one intervening gene. For synteny analysis, syntenic blocks within the grape genome, as well as between grape and *Arabidopsis* genomes, were downloaded from the Plant Genome Duplication Database [57] and those containing grape and *Arabipidopsis ZML*, *PPD* and *JAZ* genes were identified.

### Expression Analysis of Grape *TIFY* Genes

Affymetrix grape microarray data were downloaded from ArrayExpress [58] and PLEXdb [59] databases. A total of 12 experiments were used for our gene expression analyses (Table S1). For each microarray experiment, the methods utilized for normalization and to adjust background, as well as detection calls, P-value calculation and adjustment have been described previously [60]. Genes with adjusted p-values (FDR) less than 0.05 were considered to be differentially expressed genes. Hierarchical clustering of the expression profiles of these grape *TIFY* genes was performed using dChip [61].

### Plant Material

Grape tissue utilized in this research was collected from Chinese wild *Vitis pseudoreticulata* ‘Hunan-1’, which had been maintained in the grape germplasm resource orchard of Northwest A&F University, Yangling, China (34°20′, 108°24′E).

When shoots of vines were 25–35 cm in length, the third to fifth fully expanded young grapevine leaves beneath the apex were selected for hormone treatments. Hormone treatments were conducted by spraying leaves with 0.5 g/L ethylene or 50 µM MeJA followed by sampling at 0, 0.5, 1, 3, 6, 12, 24 and 48 h post-treatment. Grape leaves sprayed with water were collected as a control.

### Quantitative Real-time RT-PCR Analysis

Total RNA from grape was extracted from leaf tissues of Chinese wild *V. pseudoreticulata* ‘Hunan-1’using an improved SDS/phenol method described previously [62]. First-strand cDNA was synthesized from 1 µg DNase-treated total RNA using a mixture of PolydT and random hexamers (PrimeScript™ RTase, TaKaRa Biotechnology, Dalian, Liaoning, China). Gene-specific primers were designed for five selected grape *TIFY* genes, as well as *VvCOI1* (Table S2). Quantitative real-time PCR analysis was conducted using SYBR green (Takara Biotechnology) with an IQ5 real-time PCR machine (Bio-Rad, Hercules, CA, USA). Each reaction was carried out in triplicate with a reaction volume of 25 µl. Cycling parameters were 95°C for 30 s, followed by 40 cycles of 95°C for 5 s and 60°C for 30 s. For dissociation curve analysis, a program including 95°C for 15 s, followed by a constant increase from 60°C to 95°C, was included after the PCR cycles. The grape *Actin1* transcript (GenBank Accession number AY680701) was amplified with primers F (5′-GAT TCT GGT GAT GGT GTG AGT-3′) and R (5′-GAC AAT TTC CCG TTC AGC AGT-3′) as an internal control. Relative expression levels were analyzed using the IQ5 software and the normalized-expression method. A one-sided paired *t* test was performed using SigmaPlot 11.0 (Ashburn, VA, USA) to assess significant differences between the negative control and each treatment.

## Supporting Information

Table S1 Details of publicly available grape array datasets and *TIFY* expression profiles.
(XLS)

Table S2 Primers utilized for qRT-PCR analysis.
(DOC)
