# Correction: Genome-Wide Identification and Analysis of the *TIFY* Gene Family in Grape

**DOI:** 10.1371/annotation/c9d9d321-b821-4060-8bf9-ff181229fea7

**Published:** 2013-12-13

**Authors:** Yucheng Zhang, Min Gao, Stacy D. Singer, Zhangjun Fei, Hua Wang, Xiping Wang

The details of the publications describing the data employed for the heat map in Figure 5 were omitted from the supplementary information in the published article. The authors would like to apologize for this omission and acknowledge the work carried out by the different groups that described the datasets by replacing the supplementary Table S1 by the one provided here which includes the details of the authors and publications: http://www.plosone.org/corrections/pone.0044465.001.cn.xls

