# Supplementary material for: Genome-Wide Identification and Analysis of the TIFY Gene Family in Grape
Source: PLoS One. 2012 Sep 11;7(9):e44465. doi: 10.1371/journal.pone.0044465 (PMC3439424; doi:10.1371/journal.pone.0044465)
Supplement: Table S2 — Primers utilized for qRT-PCR analysis. (DOC) [file pone.0044465.s002.doc]

**Table S2.**

| **Gene** | **Primers for Real-time PCR** | |
| --- | --- | --- |
|  | Forward Primer (5'–3') | Reverse Primer (5'–3') |
| VvPPD1 | AAAGGAACGGTGGAACCAATAGAA | CCCGCGAGGATGTCAAAATG |
| VvPPD2 | AACAAATCTCAGGCGATCCAGC | TCCGAGTCAAAGGGACACGATG |
| VvJAZ4 | TTCAGGAAATCGGCAACAACAGA | CCCTTGGCGGCTAATAGCATG |
| VvJAZ9 | TTTACCGGGCAGAGAGCGCC | GATTCGGGCGTGCCGTTTCC |
| VvZML4 | TGCTTTTGGGAGGGCGTGAT | TGAGCAACCTCTTTTCGCACAGT |
| VvCOI1 | CGGGGATGCCTGGAGATAGAA | CCTGAACGGAGGTACAGAGCGA |
